# MicroRNAs with prognostic significance in bladder cancer: a systematic review and meta-analysis

**DOI:** 10.1038/s41598-017-05801-3

**Published:** 2017-07-17

**Authors:** Yongpeng Xie, Xin Ma, Luyao Chen, Hongzhao Li, Liangyou Gu, Yu Gao, Yu Zhang, Xintao Li, Yang Fan, Jianwen Chen, Xu Zhang

**Affiliations:** 1 Department of Urology/State Key Laboratory of Kidney Diseases, Chinese PLA General Hospital/PLA Medical School, Beijing, People’s Republic of China; 2 Medical School, Nankai University, Tianjin, People’s Republic of China; 3 Department of Urology, First Affiliated Hospital of Nanchang University, Nanchang, People’s Republic of China

## Abstract

The aim of this study was to systematically review articles that investigated the prognostic significance of different microRNAs in bladder cancer (BC). We systematically searched PubMed, Web of Science, and Embase to identify relevant studies until March 2016. After screening, 26 studies that involved 2753 patients were included. Results suggested that many miRs expression aberration may predict prognosis in patients with BC. There are six miRs (miR-21, miR-143, miR-155, miR-200, miR-214, and miR-222) were reported by at least two studies, and we performed meta-analysis in the corresponding studies. Accordingly, we found that high miR-21 expression was associated with poor overall survival [OS; hazard ratio (HR) = 3.94, 95% CI 2.08–7.44]. High miR-143 expression was associated with poor progression-free survival (PFS; HR = 3.78, 95% CI 1.61–8.89). High miR-155 expression was associated with poor PFS (HR = 8.10, 95% CI 2.92–22.48). High miR-222 expression was associated with poor OS (HR = 3.39, 95% CI 1.10–10.41). Meanwhile, low miR-214 expression was correlated with poor RFS(HR = 0.34, 95% CI 0.22–0.53). Our comprehensive systematic review concluded that microRNAs, particularly miR-21, miR-143, miR-155, miR-214, and miR-222, could serve as meticulous follow-up markers for early detection of progression or recurrence and even useful therapeutic targets for the treatment in patients with BC.

## Introduction

Among urological cancers, bladder cancer (BC) is the leading cause of death, with an estimated 76,960 new cases and 16,390 deaths in the United States in 2016 alone^1^. BC is highly heterogeneous, and its two major subsets are non-muscle-invasive BC (NMIBC) and muscle-invasive BC (MIBC)^2^. Approximately 70% of BC patients have NMIBC at first diagnosis; however, about 50%–70% of them will relapse and roughly 10%–20% will progress to MIBC^3^. MIBC, which could rapidly progress and metastasize, is correlated with a high mortality despite the improved therapeutic strategies at the moment^4^. In this regard, prediction models identifying patients with unfavorable prognosis, who may benefit from early systematic therapy, are greatly needed. Based on clinicopathological parameters, the currently used system seems inferior in accurately predicting the prognosis of BC patients with diverse and complicated tumor backgrounds^5^. Therefore, novel biomarkers that can stratify patients with poor prognosis when used alone or in combination with other clinicopathological parameters must be identified to precisely guide clinical decisions.

The detecting technique of MicroRNA (miR) molecules is an available, novel approach to evaluate tumor prognosis; hence, miRs constitute an attractive biomarker source for cancer research^6^. MiRs are small non-coding RNAs (~22 nucleotide) transcribed from DNA into RNA hairpins. MiRs post-transcriptionally regulate gene expression by binding to the 3′-UTR of target mRNAs, resulting in target mRNA degradation or inhibition of their translation^7^. MiRs are involved in a variety of biological functions and in the majority of known hallmarks of cancer, including initiation, development, and metastasis^8, 9^. Moreover, many studies have suggested that miRs have a prognostic value in several human cancers, such as colorectal^10^, breast^11^, lung^12^, and ovarian^13^ cancers. Numerous miR studies that have focused on BC have recently been conducted in the fields of outcome prediction and potential therapeutic targets. Many studies have suggested the prognostic significance of miRs in patients with BC^14–17^. In this study, we perform systematic review and meta-analysis to further increase statistical power, improve clinical translation, and comprehensively investigate the prognostic value of different miRs in patients with BC.

## Results

### Search Results

**Figure 1:**
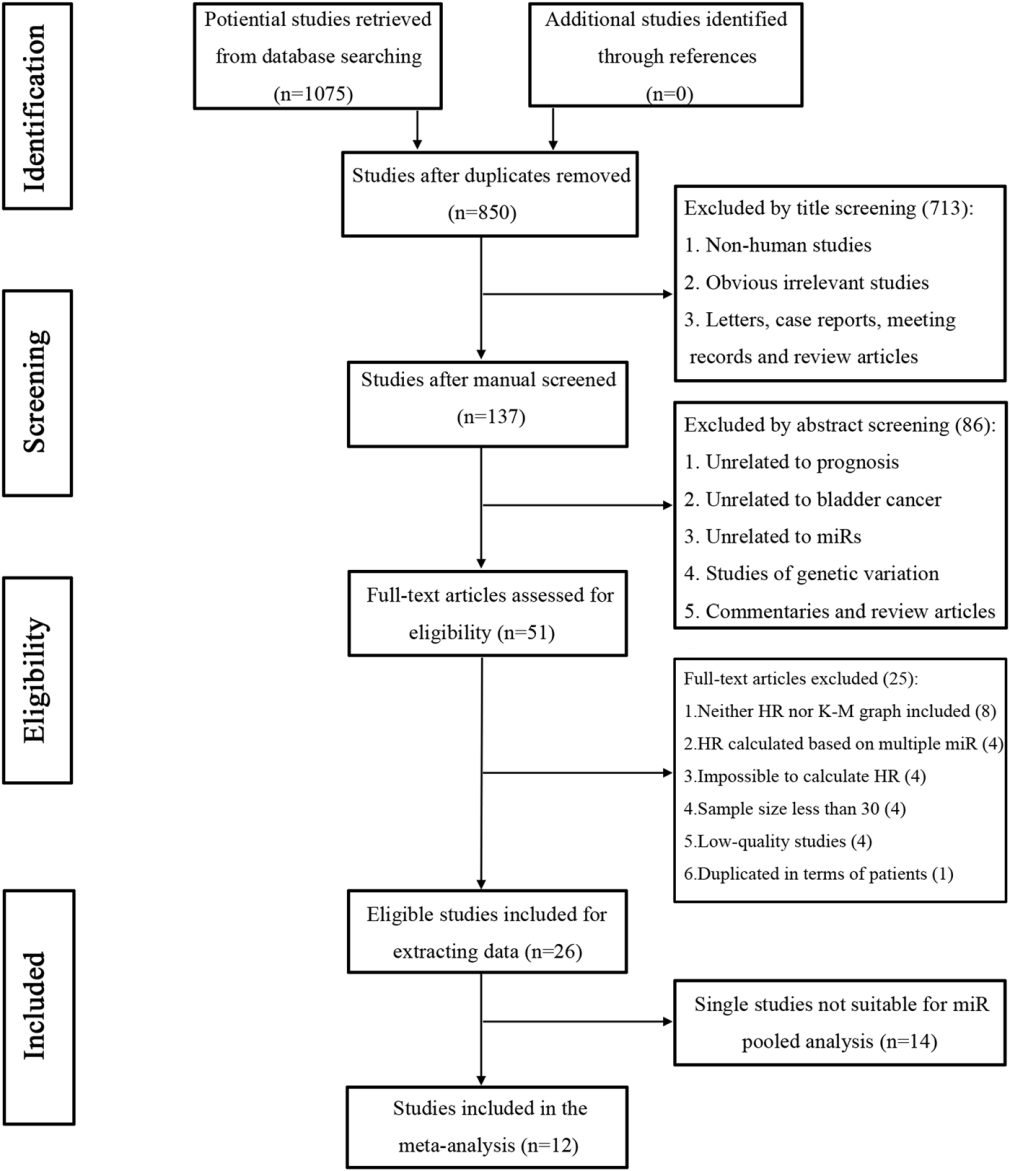
Flowchart of study selection. miR: microRNA; HR: hazard ratio.

A total of 1075 articles were retrieved from the primary literature search. A total of 225 duplicate reports were excluded. Accordingly, after screening the titles and abstracts, 799 articles were excluded because they were found to be non-human studies, genetic variation studies, letters, case reports, reviews, commentaries, and other obvious irrelevant studies. The remaining articles were viewed in full text. After a careful review of the potential articles, 26 articles were included in this study and used for data extraction (Table 1 and Fig. 1). Six prognostic miRs (miR-21, miR-143, miR-155, miR-200, miR-214, and miR-222) in BC repeatedly appeared in the included studies and triggered a meta-analysis. Only 12 articles evaluating the relationship between six specific miRs and BC prognosis satisfied the criteria for meta-analysis^14, 16, 18–27^. Figure 1 shows a flowchart of the study selection process.

**Table 1:** The main characteristics of eligible studies.

Study (year)	miR	Population	Study design	Stage	Case number	Gender (M/F)	Follow up (month)	Detecting method	Detected sample	Cut-off	Survival outcome	HR availability	Adjusted	Quality score^a^
Veerla 2009	452	Sweden	R cohort study	Ta-T3	34	NR	> 50	ISH	Tissue	Upper tertile	OS	Report	NR	6
	452*	Sweden	R cohort study	Ta-T3	34	NR	> 50	ISH	Tissue	Upper tertile	OS	Report	NR	
Dyrskjøt 2009	133b	Denmark	R cohort study	Ta-T4	106	81/25	~90	qRT-PCR	Tissue	ROC curve	PFS	Report	Yes	7
	518c*	Denmark	R cohort study	Ta-T4	106	81/25	~90	qRT-PCR	Tissue	ROC curve	PFS	Report	Yes	
	129	Denmark	R cohort study	Ta-T4	106	81/25	~90	qRT-PCR	Tissue	ROC curve	PFS	Report	Yes	
	29c	Denmark	R cohort study	Ta-T4	106	81/25	~90	qRT-PCR	Tissue	ROC curve	PFS	Report	Yes	
Wang 2012	100	China	R cohort study	Ta-T4	126	87/39	36	qRT-PCR	Tissue	Median	OS,PFS	Report	Yes	7
Yun 2012	200	Korea	P cohort study	Ta-T1	138	NR	7–76	qRT-PCR	Urine cell-free	ROC curve	RFS	Report	Yes	8
Zaravinos 2012	21	Greece	R cohort study	Ta-T4	77	68/9	~50	qRT-PCR	Tissue	Median	OS,RFS	Report	Yes	8
	210	Greece	R cohort study	Ta-T4	77	68/9	~50	qRT-PCR	Tissue	Median	OS	Report	Yes	
	387	Greece	R cohort study	Ta-T4	77	68/9	~50	qRT-PCR	Tissue	Median	OS,RFS	Report	Yes	
Puerta-Gil 2012	222	Spain	P cohort study	Ta-T4	113	101/12	36	qRT-PCR	Tissue	Median	OS,CSS, RFS,PFS	SC	NR	6
	143	Spain	P cohort study	Ta-T4	113	101/12	36	qRT-PCR	Tissue	Median	RFS,PFS	SC	NR	
Kim 2013	214	Korea	R cohort study	Ta-T1	138	110/28	16–82	qRT-PCR	Urine cell-free	Median	RFS	Report	Yes	7
Wang 2013	31	China	R cohort study	Ta-T4	126	87/39	36	qRT-PCR	Tissue	Median	OS,PFS	Report	Yes	8
Rosenberg 2013	29c*	Israel	R cohort study	Ta-T1	75	NR	53	ISH	Tissue	Upper tertile	PFS	SC	NR	6
Ratert 2013	141	Germany	R cohort study	Ta-T4	40	32/8	> 100	qRT-PCR	Tissue	Median	OS	SC	NR	6
	205	Germany	R cohort study	Ta-T4	40	32/8	> 100	qRT-PCR	Tissue	Median	OS	SC	NR	
Pignot 2013	9	France	R cohort study	T2-T4	72	58/15	15	qRT-PCR	Tissue	Median	OS,RFS	SC	NR	6
	182	France	R cohort study	T2-T4	72	58/15	15	qRT-PCR	Tissue	Median	OS,RFS	SC	NR	
	200	France	R cohort study	T2-T4	72	58/15	15	qRT-PCR	Tissue	Median	OS,RFS	SC	NR	
Zhang 2014	101	China	R cohort study	T1-T4	72	42/30	~80	qRT-PCR	Tissue	T/N ratio > 0.45	OS	Report	Yes	8
Zhang 2014	222	China	R cohort study	Ta-T4	97	51/46	~60	qRT-PCR	Tissue	Median	OS	Report	Yes	8
Lin 2014	26a	China	R cohort study	Ta-T4	126	80/46	40	qRT-PCR	Tissue	Median	OS,DFS	Report	Yes	9
Drayton 2014	27a	UK	P cohort study	Ta-T4	139	100/39	~96	qRT-PCR	Tissue	T/N ratio > 2	RFS,PFS	DE	NR	6
	27b	UK	P cohort study	Ta-T4	139	100/39	~96	qRT-PCR	Tissue	T/N ratio > 2	RFS,PFS	DE	NR	6
Zhang 2015	203	China	R cohort study	T2-T4	108	83/25	51.5	qRT-PCR	Tissue	ROC curve	OS,PFS	Report	Yes	9
Zhang 2015	21	China	R cohort study	T1-T4	53	35/18	~60	qRT-PCR	Tissue	T/N ratio > 6.7	OS	Report	Yes	8
Wu 2015	424	China	R cohort study	Ta-T4	124	77/47	94–144	ISH	Tissue	X-tile algorithm	OS,DFS	SC/Report	NR/Yes	6
Wang 2015	141	China	R cohort study	Ta-T4	114	86/28	43	qRT-PCR	Tissue	Median	CSS,DFS	Report	Yes	8
Wang 2015	214	China	P cohort study	T2-T4	129	NR	29	qRT-PCR	Urine cell-free	Median	OS,RFS	Report	Yes	8
Wang 2015	155	China	R cohort study	Ta-T4	102	61/41	~60	qRT-PCR	Tissue	Median	PFS	Report	Yes	8
Jiang 2015	152	China	R cohort study	Ta-T1	59	NR	~62	qRT-PCR	Serum	Median	RFS	Report	Yes	6
	148b-3p	China	R cohort study	Ta-T1	59	NR	~62	qRT-PCR	Serum	Median	RFS	Report	NR	
	3187–3p	China	R cohort study	Ta-T1	59	NR	~62	qRT-PCR	Serum	Median	RFS	Report	Yes	
	15b-5p	China	R cohort study	Ta-T1	59	NR	~62	qRT-PCR	Serum	Median	RFS	Report	NR	
	27a-3p	China	R cohort study	Ta-T1	59	NR	~62	qRT-PCR	Serum	Median	RFS	Report	NR	
	30a-5p	China	R cohort study	Ta-T1	59	NR	~62	qRT-PCR	Serum	Median	RFS	Report	NR	
Avgeris 2015	143	Greece	R cohort study	Ta-T4	133	NR	~48	qRT-PCR	Tissue	X-tile algorithm	OS,PFS	Report	NR	6
	145	Greece	R cohort study	Ta-T4	133	NR	~48	qRT-PCR	Tissue	X-tile algorithm	OS,PFS	Report	NR	
	224	Greece	R cohort study	Ta-T4	133	NR	~48	qRT-PCR	Tissue	X-tile algorithm	OS,PFS	Report	NR	
Andrew 2015	34a	USA	R cohort study	Ta-T1	229	171/58	46	ISH	Tissue	fluorescence scores 1+	RFS	Report	Yes	8
Martínez-Fernández 2015	200	Spain	R cohort study	Ta-T1	61	NR	28.8	qRT-PCR	Serum	Median	RFS	SC	NR	6
Zhang 2016	155	China	P cohort study	Ta-T1	162	126/36	51.5	qRT-PCR	Urine cell-free	ROC curve	RFS,PFS	Report	Yes	9

miR: microRNA; HR: hazard ratio; R: retrospective; P: prospective; qRT-PCR: quantities reverse transcription polymerase chain reaction; ISH: *in situ* hybridization; OS: overall survival; CSS: cancer-specific survival; RFS: recurrence-free survival; PFS: progression-free survival; DFS: disease-free survival; SC: survival curve; NR: not reported.

^a^The quality of the included studies was evaluated using the Newcastle-Ottawa scale.

### Study Characteristics

All of the included studies were recently published (2009–2016). They reported the prognostic significance of 37 different miRs in BC patients with varying tumor stages. Thirteen studies originated from China, two from Korea, two from Greece, two from Spain, one from Sweden, one from Denmark, one from Israel, one from Germany, one from France, one from the United States, and one from the United Kingdom. Most studies applied the quantitative real-time polymerase chain reaction to measure the miR expression. Four studies applied *in situ* hybridization. The miR expressions were mainly detected in the tissue samples. However, two and four studies measured miRs in serum and urine cell-free, respectively. The HR was adjusted for corresponding covariates in 18 studies using the Cox regression multivariate analysis. Table 1 summarizes the detailed information of the twenty-six included studies.

### MiRs and Prognosis

**Figure 2:**
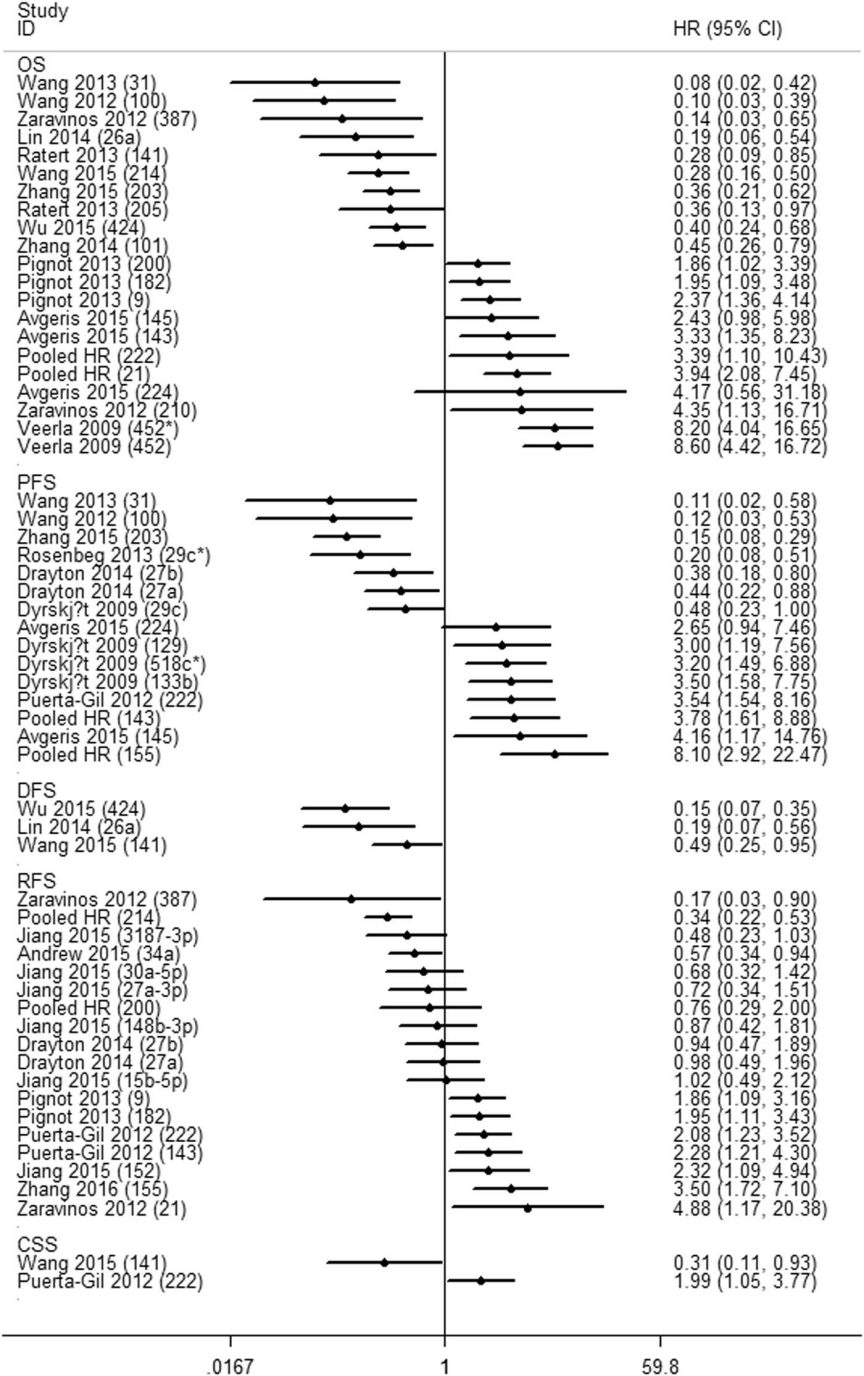
HR of miRs. The point estimate is bounded by a 95% CI (indicated by error bars), and the perpendicular line represents no increased risk for the outcome. HR: hazard ratio; CI: confidence interval; OS: overall survival; PFS: progression-free survival; RFS: recurrence-free survival; CSS: cancer-specific survival; DFS: disease-free survival. HR > 1 implied an unfavorable prognosis for the group with an elevated miR expression.

High miR-452^28^, miR-452*^28^, miR-21^16, 25^, miR-210^25^, miR-222^22, 27^, miR-9^23^, miR-182^23^, miR-143^21, 27^, miR-133b^29^, miR-518c*^29^, miR-129^29^, miR-155^14, 19^, miR-145^21^, and miR-152^30^ expressions were associated with poor prognosis. Conversely, low miR-100^31^, miR-387^25^, miR-31^32^, miR-141^33^, miR-205^33^, miR-101^34^, miR-26a^35^, miR-203^15^, miR-424^36^, miR-214^17, 24^, miR-29c*^37^, miR-27a^38^, miR-27b^38^, miR-203^15^, and miR-34a^39^ expressions were correlated with unfavorable prognosis. The miR-200^20, 23, 26^, miR-224^21^, miR-29c^29^, miR-148b-3p^30^, miR-3187-3p^30^, miR-15b-5p^30^, miR-27a-3p^30^, and miR-30a-5p^30^ expression did not show any significant association with survival outcomes (Table 2 and Fig. 2). Six miRs (i.e., miR-21, miR-143, miR-155, miR-200, miR-214, and miR-222) were assessed by at least two studies. We performed a meta-analysis in the corresponding studies.

**Table 2:** Summary of HR of miRNA expression in bladder cancer.

Study	miR	Case number		OS		CSS		RFS		DFS/PFS		Expression associates with bad prognosis
		High level	Low level	HR (95% CI)	P	HR (95% CI)	P	HR (95% CI)	P	HR (95% CI)	P	
Veerla 2009	452	11	23	8.6 (3.6–13.6)	< 0.025	—	—	—	—	—	—	High
	452*	11	23	8.2 (3.2–13.2)	< 0.025	—	—	—	—	—	—	High
Dyrskjøt 2009	133b	—	—	—	—	—	—	—	—	3.5 (1.58–7.75)^P^	0.002	High
	518c*	—	—	—	—	—	—	—	—	3.2 (1.49–6.89)^P^	0.003	High
	129	—	—	—	—	—	—	—	—	3.0 (1.19–7.56)^P^	0.02	High
	29c	—	—	—	—	—	—	—	—	0.48 (0.23–1)^P^	0.05	High
Wang 2012	100	48	78	0.10 (0.04–0.6)	0.008	—	—	—	—	0.12 (0.04–0.77)^P^	0.01	Low
Yun 2012	200	—	—	—	—	—	—	0.449 (0.239–0.842)	0.013	—	—	Low
Zaravinos 2012	21	38	39	8.40 (1.90–37.04)	0.005	—	—	4.88 (1.17–20.41)	0.03	—	—	High
	210	38	39	4.35 (1.13–16.67)	0.033	—	—	—	—	—	—	High
	387	38	39	0.14 (0.03–0.65)	0.012	—	—	0.17 (0.03–0.85)	0.031	—	—	Low
Puerta-Gil 2012	222	56	57	1.96 (1.10–3.48)	0.023	1.99 (1.05–3.76)	0.034	2.08 (1.23–3.52)	0.006	3.54 (1.54–8.18)^P^	0.003	High
	143	56	57	—	—	—	—	2.28 (1.21–4.31)	0.011	3.01 (1.06–8.59)^P^	0.039	High
Kim 2013	214	69	69	—	—	—	—	0.497 (0.254–0.974)	0.041	—	—	Low
Wang 2013	31	56	70	0.084 (0.033–0.833)	0.008	—	—	—	—	0.114 (0.039–1)^P^	0.01	Low
Rosenbeg 2013	29c*	18	57	—	—	—	—	—	—	0.2 (0.08–0.52)^P^	< 0.001	Low
Ratert 2013	141	20	20	0.28 (0.09–0.82)	0.02	—	—	—	—	—	—	Low
	205	20	20	0.36 (0.13–0.95)	0.04	—	—	—	—	—	—	Low
Pignot 2013	9	36	36	2.37 (1.36–4.15)	0.003	—	—	1.86 (1.08–3.12)	0.025	—	—	High
	182	36	36	1.95 (1.09–3.47)	0.024	—	—	1.95 (1.11–3.43)	0.021	—	—	High
	200	36	36	1.86 (1.02–3.39)	0.043	—	—	1.93 (1.09–3.39)	0.023	—	—	High
Zhang 2014	101	46	26	0.451 (0.237–0.735)	0.028	—	—	—	—	—	—	Low
Zhang 2014	222	48	49	6.17 (2.33–10.39)	< 0.001	—	—	—	—	—	—	High
Lin 2014	26a	56	70	0.185 (0.088–0.762)	0.01	—	—	—	—	0.192 (0.0891–0.745)^D^	0.01	Low
Drayton 2014	27a	70	69	—	—	—	—	0.98 (0.49–1.96)	0.96	0.44 (0.22–0.88)^P^	0.02	Low
	27b	63	76	—	—	—	—	0.94 (0.47–1.90)	0.87	0.38 (0.18–0.80)^P^	0.01	Low
Zhang 2015	203	79	29	0.359 (0.209–0.616)	< 0.001	—	—	—	—	0.154 (0.082–0.288)^P^	< 0.001	Low
Zhang 2015	21	28	25	3.32 (1.16–4.74)	0.018	—	—	—	—	—	—	High
Wu 2015	424	53	71	0.40 (0.24–0.69)	0.001	—	—	—	—	0.152 (0.066–0.350)^D^	< 0.001	Low
Wang 2015	141	54	60	—	—	0.314 (0.108–0.946)	0.039	—	—	0.492 (0.254–0.954)^D^	0.036	Low
Wang 2015	214	64	65	0.282 (0.160–0.495)	< 0.001	—	—	0.264 (0.149–0.468)	< 0.001	—	—	Low
Wang 2015	155	52	50	—	—	—	—	—	—	7.7 (1.4–14.7)^P^	0.009	High
Jiang 2015	152	29	30	—	—	—	—	2.324 (1.093–4.940)	0.028	—	—	High
	148b-3p	29	30	—	—	—	—	0.872 (0.419–1.812)	0.713	—	—	Low
	3187–3p	29	30	—	—	—	—	0.483 (0.227–1.028)	0.059	—	—	Low
	15b-5p	29	30	—	—	—	—	1.023 (0.494–2.120)	0.951	—	—	High
	27a-3p	29	30	—	—	—	—	0.721 (0.344–1.510)	0.386	—	—	Low
	30a-5p	29	30	—	—	—	—	0.677 (0.323–1.419)	0.302	—	—	Low
Avgeris 2015	143	—	—	3.329 (1.346–8.236)	0.009	—	—	—	—	5.990 (1.351–26.55)^P^	0.018	High
	145	—	—	2.426 (0.985–5.976)	0.054	—	—	—	—	4.164 (1.178–14.80)^P^	0.027	High
	224	—	—	4.168 (0.557–31.183)	0.164	—	—	—	—	2.654 (0.944–7.460)^P^	0.064	High
Andrew 2015	34a	63	166	—	—	—	—	0.57 (0.34–0.93)	0.029	—	—	Low
Martínez-Fernández 2015	200	31	30	—	—	—	—	0.49 (0.25–0.97)	0.041	—	—	Low
Zhang 2016	155	130	32	—	—	—	—	3.497 (1.722–7.099)	0.001	9.466 (1.210–74.066)^P^	0.032	High

miR: microRNA; HR: hazard ratio; CI: confidence interval; OS: overall survival; CSS: cancer-specific survival; RFS: recurrence-free survival; PFS: progression-free survival;

DFS: disease-free survival; —: not reported.

^D^DFS; ^P^PFS.

**Figure 3:**
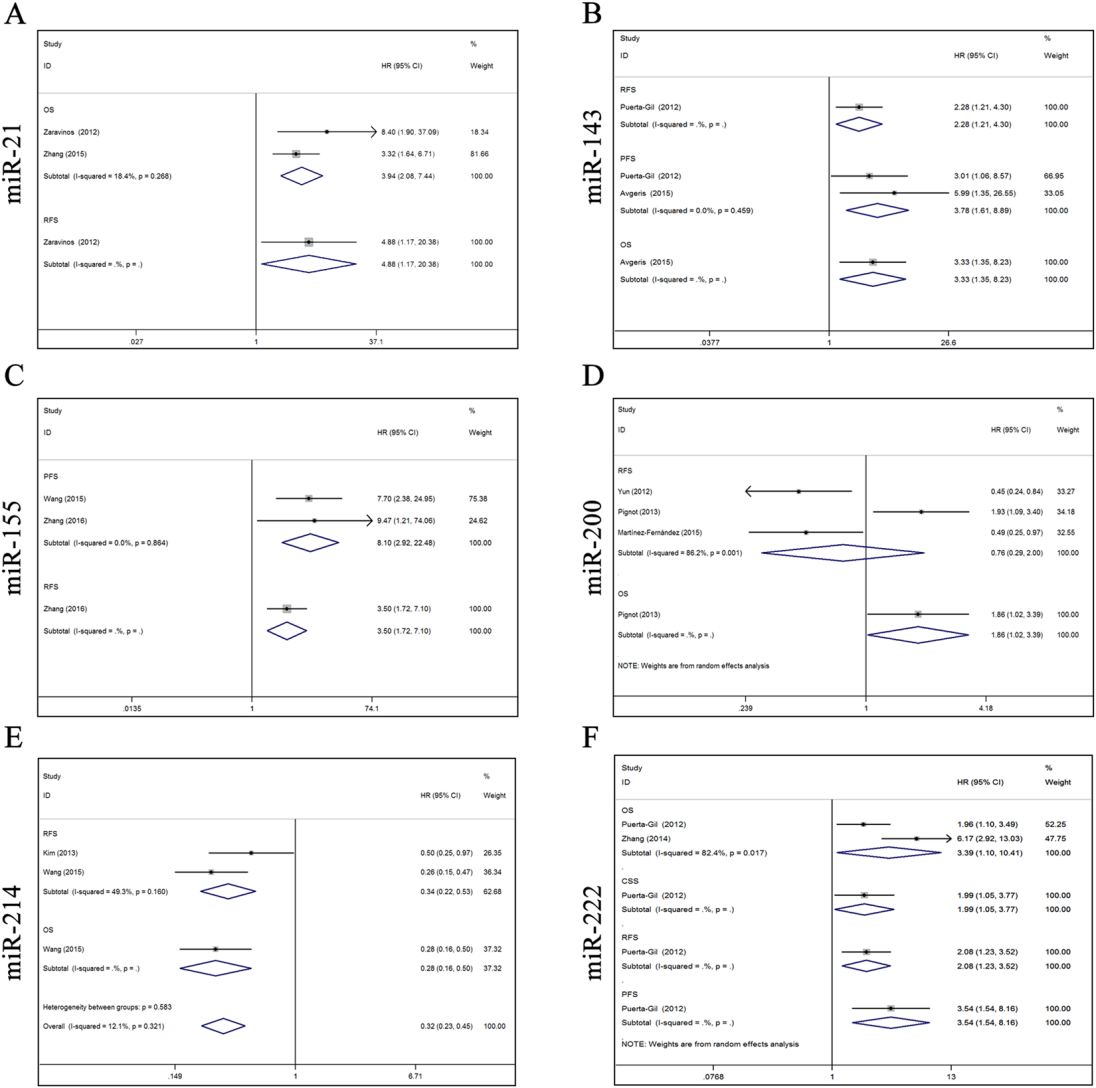
Forest plots of studies evaluating HR of six aberrant miRs expression. (**A**) miR-21, OS, RFS; (**B**) miR-143, RFS, PFS, OS; (**C**) miR-155, PFS, RFS; (**D**) miR-200, RFS, OS; (**E**) miR-214, RFS, OS; (**F**) miR-222, OS, CSS, RFS, PFS. HR: hazard ratio; OS: overall survival; PFS: progression-free survival; RFS: recurrence-free survival; CSS: cancer-specific survival. HR > 1 implied an unfavorable prognosis for the group with an elevated miR expression.

Two studies described the association of miR-21 with survival outcomes in BC, of which two reported overall survival (OS)^16, 25^ and one reported recurrence-free survival (RFS)^25^. Next, we conducted a meta-analysis on the miR-21 expression and OS relationship. The results showed that high miR-21 expression was correlated with poor OS [a fixed-effect model, hazard ratio (HR), 3.94; 95% CI: 2.08–7.44; p < 0.001; *I* ^2^ = 18.4%, p = 0.268]. Zaravinos *et al*.^25^ also reported shorter RFS in BC patients with an elevated level of miR-21 (HR, 4.88; 95% CI: 1.17–20.41; p = 0.03) (Fig. 3A).

Two studies included survival outcomes for miR-143 in BC, of which two had progression-free survival (PFS)^21, 27^ data, one had OS^21^ data, and one contained RFS^27^ data. When we performed a meta-analysis on the relationship of miR-143 expression and PFS, the results showed that a higher miR-143 expression was predictive of shorter PFS (a fixed-effect model, HR, 3.78; 95% CI: 1.61–8.89; p = 0.002; *I* ^2^ = 0, p = 0.459). Avgeris *et al*.^21^ and Puerta-Gil *et al*.^27^ also reported shorter OS (HR, 3.33; 95% CI: 1.35–8.24; p = 0.009) and RFS (HR, 2.28; 95% CI: 1.21–4.31; p = 0.011) in BC patients with a higher miR-143 levels (Fig. 3B).

Two studies addressed the role of miR-155 in survival outcomes of BC patients, of which two reported PFS^14, 19^, and one reported RFS^14^. Then, we conducted a meta-analysis on the miR-155 expression and PFS relationship. This indicated that elevated miR-155 expression was correlated with poor PFS (a fixed-effect model, HR, 8.10; 95% CI: 2.92–22.48; p < 0.001; *I* ^2^ = 0, p = 0.864). Additionally, Zhang *et al*.^14^ also found shorter RFS in BC patients with an higher miR-155 level (HR, 3.50; 95% CI: 1.72–7.10; p = 0.001) (Fig. 3C).

Three studies investigated the association between miR-200 expression and the prognosis of BC patients, of which three focused on RFS^20, 23, 26^ and one focused on OS^23^. A meta-analysis was performed on the relationship of miR-200 expression and RFS. The results indicated that aberrant miR-200 expression was not related to RFS (a random-effect model, HR, 0.76; 95% CI: 0.29–2.00; p = 0.580; *I* ^2^ = 86.2%, p = 0.001). However, Pignot *et al*.^23^ reported that an increased miR-200 level was correlated with poor OS (HR, 1.86; 95% CI: 1.02–3.39; p = 0.043) (Fig. 3D).

Two studies addressed the relationship between miR-214 expression and survival outcomes in BC, of which two contained RFS^18, 24^ data, and one contained OS^18^ data. A meta-analysis was conducted on the miR-214 expression and RFS relationship. We found that low miR-214 expression was predicted poor RFS (a fixed-effect model, HR, 0.34; 95% CI: 0.22–0.53; p < 0.001; *I* ^*2*^ = 49.3%, p = 0.160). In addition, Wang *et al*.^18^ also found that a shorter OS in BC patients with an decreased level of miR-214 (HR, 0.28; 95% CI: 0.16–0.50; p < 0.001) (Fig. 3E).

For miR-222, two studies reported OS^22, 27^, one reported CSS^27^, one reported RFS^27^, and one reported PFS^27^. We performed a meta-analysis on the miR-222 expression and OS relationship. The results suggested that increased miR-222 expression tended to occur in patients with poor OS (a random-effect model, HR, 3.39; 95% CI: 1.10–10.41; p = 0.033; *I* ^2^ = 82.4, p = 0.017). Additionally, Puerta-Gil *et al*.^27^ found that a poor CSS (HR, 1.99; 95% CI: 1.05–3.77; p = 0.034), RFS (HR, 2.08; 95% CI: 1.23–3.52; p = 0.006), and PFS (HR, 3.54; 95% CI: 1.54–8.16; p = 0.003) in BC patients with an elevated miR-222 level (Fig. 3F).

Considering that few studies were included in this meta-analysis, the publication bias evaluated by Begg’s and Egger’s test was not considered necessary.

## Discussion

We performed comprehensive systematic review and meta-analysis of the current literature on BC in response to the need for independent prognostic molecular markers that are readily assayable before, during, and/or after BC treatment. A total of 26 studies involving 2753 patients were analyzed to evaluate the relationship between the miRs and the BC prognosis in our study. Accordingly, 37 different miRs involved in the survival outcomes of patients with BC were compared. This study aimed to identify the miRs associated with BC prognosis, which could be further validated in future studies and eventually evaluated before the treatment, thereby improving BC management.

This study found that high miR-452^28^, miR-452*^28^, miR-21^16, 25^, miR-210^25^, miR-222^22, 27^, miR-9^23^, miR-182^23^, miR-143^21, 27^, miR-133b^29^, miR-518c*^29^, miR-129^29^, miR-155^14, 19^, miR-145^21^, and miR-152^30^ expressions were correlated with poor prognosis. In comparison, low miR-100^31^, miR-387^25^, miR-31^32^, miR-141^33^, miR-205^33^, miR-101^34^, miR-26a^35^, miR-203^15^, miR-424^36^, miR-214^17, 24^, miR-29c*^37^, miR-27a^38^, miR-27b^38^, miR-203^15^, and miR-34a^39^ expressions were correlated with unfavorable prognosis (Table 2 and Fig. 2).

Our study systematically assessed the role of 37 different miRs in BC prognosis. However, most of the miRs were investigated only by a single study, and only six miRs (miR-21, miR-143, miR-155, miR-200, miR-214, and miR-222) were identified by at least two studies. Therefore, we conducted the meta-analysis on these six miRs to determine a pooled conclusion. We found that high miR-21 expression was associated with poor OS. High miR-143 expression was associated with poor PFS. High miR-155 expression was associated with poor PFS. Meanwhile, high miR-222 expression was associated with poor OS. Low miR-214 expression was correlated with poor RFS, and MiR-200 expression was not related to the RFS in the pooled analysis. Pignot *et al*.^23^ reported that an elevated miR-200 level was associated with a shorter OS. But it was important to note that the study was small (n = 72), had limited power, could lead to a premature result, and was not adjusted for other associated variables (covariates) that could also affect the survival outcome. Therefore, more multicenter prospective studies with large scale and long-term follow-up are needed to obtain a more persuasive conclusion.

MiR-21 is one of the most extensively studied cancer-related miRs; it is an abnormal expression in most cancers functioning as oncogene^40–43^. Zhou *et al*.^44^ published a meta-analysis evaluating the prognostic value of miR-21 in various cancers. They pooled 63 published studies and found that the increased miR-21 expression predicted worse OS in cancers. This finding is consistent with our results in BC. Some researchers explored the possible miR-21 mechanism in BC. Zhang *et al*.^16^ suggested that miR-21 through the maspin expression down-regulation up-regulated the VEGF-C expression, thereby increasing tumor growth, migration, and invasion in BC. Additionally, Zaravinos *et al*.^25^ demonstrated that miR-21 repressed the tumor suppressors, PTEN and PDCD4, to enhance angiogenesis, tumor cell proliferation, EMT, and metastasis activation in BC. MiR-143 is located in chromosome 5q33, which is a well-known fragile site in human genome and highly co-expressed with miR-145 in BC^45, 46^. Many studies have demonstrated the tumor suppressor role of miR-143 in BC. MiR-143 can suppress cell proliferation and migration and promote apoptosis in BC by inhibiting PI3K/Akt and MAPK signaling and targeting AKT^47^, KRAS^48^, ERK5^49^, and PAI-1^45^. Intriguingly, miR-143 has been found to be up-regulated in aggressive BC, and that the case of patients with evaluated miR-143 is associated with worse prognosis. This finding seemed to be contrary to the tumor suppressor functions of miR-143. Many studies have also observed this interesting outcome^21, 23, 27, 29^. However, the definite mechanism elucidating it remains unclear and needs further studies. A large number of studies confirmed the oncogene function of miR-155 in various cancers, including renal, thyroid, hematological, pancreatic, and bladder cancers^50–54^. High miR-155 expression has recently been correlated with BC recurrence and progression. Some studies might provide potential evidence linking the miR-155 expression and BC. Zhang *et al*.^14^ suggested that miR-155 overexpression promoted tumor cell growth via Wnt/β-catenin signaling activation, which is also a vital pathway in the BC tumorigenesis. In addition, Wang *et al*.^19^ reported that the oncogenic properties of miR-155 can be attributed to its antiapoptotic function through a blockade of caspase-3 activity or suppression of proapoptotic genes, such as TP53BP1, and promoted cell proliferation by down-regulating the SOCS1 gene, or activated PKB signaling via downregulation of tumor suppressors, including PTEN, PDCD4, and SHIP1. The role of miR-214 acting as an oncogene or a tumor suppressor is quite distinctive in different cancer types. MiR-214, serving as an oncogene, has been found in many human cancers, such as nasopharyngeal, gastric, and ovarian cancers as well as malignant melanomas^55–58^. Nevertheless, miR-214, serving as a tumor suppressor, has been found in several other cancers, such as cervical, breast, liver, esophagus, and bladder cancers^17, 59–62^. The functional discrepancies of miR-214 in different cancer types may be derived from its varied target genes or distinction among tissue types and cellular environments. Wang *et al*.^17^ showed that miR-214 decreased cellular proliferation, migration, and invasion and simultaneously increased apoptosis, suggesting that miR-214 functions a tumor suppressor in BC. The tumor-suppressive role of miR-214 might explain the unfavorable prognosis of BC patients with low miR-214 expression. High miR-222 expression has been observed in many human cancers, such as glioblastoma, prostate, colorectal, and pancreatic cancers^63–66^, suggesting that miR-222 might play a vital role in carcinogenesis. Puerta-Gil *et al*.^27^ recently found that high miR-222 expression is correlated with more advanced tumor stage and grade in BC, indicating its important role in tumorigenesis and metastasis. Moreover, Zhang *et al*.^22^ obtained the same conclusion in Asian patients with BC. Calderaro *et al*.^67^ explored the possible molecular mechanism of miR-222 in BC. They found that miR-222 decreased the tumor suppressor, PTEN, which is considered to enhance angiogenesis, tumor cell proliferation, EMT, and metastasis activation in BC. This finding might account for the poor prognosis of BC patients with low miR-222 expression.

Aside from the abovementioned miRs, the current study also systematically investigated the relationship between other miRs and BC prognosis. Figure 2 and Table 3 summarize the relation with prognosis and the possible role of other miRs in BC progression.

**Table 3:** Summary of possible role and potential mechanism of miRs entered this study in bladder cancer.

microRNA	Role	Mechanism	Reference
miR-155	Proto-oncogene	**Zhang:** miR-155 overexpression promotes some tumor cell growth via Wnt/β-catenin signaling activation which is also a vital pathway in bladder cancer tumorigenesis.	14, 19
		**Wang:** Oncogenic properties of miR-155 are attributed to its antiapoptotic function through a blockade of caspase-3 activity or suppression of proapoptotic genes such as TP53BP1 and promote cell proliferation by down-regulating the SOCS1 gene, or activate PKB signaling via downregulation of tumor suppressors, including PTEN, PDCD4, and SHIP1.	
miR-203	Tumor suppressor	miR-203 simultaneously suppressed antiapoptotic factors Bcl-w and Survivin.	15
miR-21	Proto-oncogene	**Zhang:** miR-21 functions through regulation of maspin and VEGF-C, suggesting a miR-21/maspin/VEGF-C pathway in bladder cancer.	16, 25
		**Zaravinos:** miR-21 represses the tumor suppressors PTEN deleted on chromosome 10, tropomyosin 1 and the PDCD4.	
miR-424	Tumor suppressor	miR-424 regulates multiple cellular biological behaviors, such as retarding growth, inducing apoptosis, and reducing invasion, by directly targeting EGFR in bladder cancer.	36
miR-214	Tumor suppressor	**Wang:** miR-214 could exert tumor-suppressive effects in bladder cancer by directly down-regulating oncogene PDRG1.	17, 24
		**Kim:** miR-214 could be related to the inhibition of angiogenesis, to cell proliferation, and to tumor recurrence.	
miR-152	Proto-oncogene	miR-152 acts through upregulation of DNA hypermethylation in bladder cancer.	30
miR-27a-3p	Tumor suppressor	miR-27a-3p as a target of mutant p53–273 H and uncovered a novel mutant p53–273 H/miR-27a-3p/EGFR pathway which played an important role in tumorigenesis.	30
miR-143	Tumor suppressor	miR-143 can suppress cell proliferation and migration as well as promote apoptosis in bladder cancer by inhibiting PI3K/Akt and MAPK signaling.	21
miR-224	Proto-oncogene	The upregulation of miR-224 levels has been observed to promote cell migration and tumor growth by targeting the tumor suppressors.	21
miR-34a	Tumor suppressor	miR-34a expression can inhibit cell migration and invasion by antagonizing Notch1 signaling.	39
miR-101	Tumor suppressor	Abnormal down-regulation of miR-101 could frequently lead to the overexpression of EZH2 in cancer, which increased cell proliferation in bladder cancer cells and retarded transition of G phase to S phase.	34
miR-222	Proto-oncogene	miR-222 decreased the tumor suppressor PTEN, which was considered to enhance angiogenesis, tumor cell proliferation, EMT and activation of metastasis in bladder cancer.	67
miR-26a	Tumor suppressor	miR-26a functions through regulation of HMGA1 in bladder cancer.	35
miR-29c	Tumor suppressor	miR-29c regulates the apoptotic protein MCL1 and thereby regulating apoptosis as well as DNA de novo methyltransferases DNMT3A and DNMT3B, key enzymes that are frequently up-regulated in cancer.	29
miR-210	Proto-oncogene	miR-210 over expression activates VEGF and leads to the formation of capillary structures under hypoxic conditions during the early steps of tumor development.	25
miR-200	Tumor suppressor	Higher levels of miR-200 might inhibit EMT and prevent non-muscle invasive bladder cancer recurrence through the silencing of various target genes.	26
miR-27a	Tumor suppressor	miR-27a functions through regulation of cystine/glutamate exchanger SLC7A11 in bladder cancer.	38
miR-129	Proto-oncogene	miR-129 simultaneously repressed the tumor suppressors SOX4 and GALNT1 in bladder cancer.	29
miR-29c*	Tumor suppressor	miR-29c* acts through downregulation of DNA methyltransferases as well as upregulation of demethylating genes to keep the normal methylation pattern.	37
miR-9	Proto-oncogene	miR-9 directly targeted the CDH-1 gene encoding E-cadherin, a regulator of EMT, considered to be an important initiating step for tumor metastasis.	23

TP53BP1: tumor protein p53 binding protein 1; SOCS1: suppressor of cytokine signaling 1; PKB: protein kinase B; PTEN: phosphatase and tensin homolog; PDCD4: programmed cell death 4; VEGF-C: vascular endothelial growth factor-C; EGFR: epidermal growth factor receptor; PDRG1: p53 and DNA-damage regulated 1; EZH2: enhancer of zeste homologue 2; HMGA1: high mobility group AT-hook 1; MCL1: myeloid cell leukemia 1; DNMT3A: DNA-methyltransferase 3 alpha; DNMT3B: DNA-methyltransferase 3 beta; EMT: epithelial-to-mesenchymal transition; SLC7A11: solute carrier family 7 member 11; SOX4: SRY-box 4; GALNT1: polypeptide N-acetylgalactosaminyltransferase 1; CDH1: cadherin 1.

Accordingly, several limitations should be pointed out in the interpretation of the results of the current study. First, 26 involving 2753 patients were included in the systematic review. However, most of them investigated diverse miRs. Only six miRs (miR-21, miR-143, miR-155, miR-200, miR-214, and miR-222) were assessed by at least two studies that used different survival outcome assessments. Therefore, only two or three records were eligible in most of the meta-analyses in the current study. Furthermore, more multicenter prospective studies should be performed to verify our results and make a more mature conclusion. The publication bias was not evaluated because only few studies were included in this meta-analysis. The lack of analysis might have affected the interpretation of the results and make them less reliable. Second, error may have occurred because of inaccurate readings even if three independent reviewers extracted the data and the HR extrapolation method from a Kaplan–Meier graph was previously validated^68^. Therefore, the extrapolated HRs might be less dependable compared with the reported statistics. Third, the marked heterogeneity of studies was observed in some analyses. The heterogeneity of the pooled analysis might have been caused by several differences among the studies, including the baseline characteristics of the patients (i.e., study size, age, gender, ethnicity, and tumor stage), detecting methods, detecting sample, cut-off value, HR source, adjusted covariates, and follow-up duration. Finally, positive results were more likely to be published in most studies, whereas studies with negative results were often rejected or less assessable, which could compromise the validity of such analyses^69^. Urine markers have recently become more desirable than tissue or serum markers because urine is more convenient and less invasive to collect. Additionally, urine markers can be evaluated before, during, and/or after surgery and then monitored throughout a patient’s life. More well-conducted and appropriately designed studies are thus needed to establish the prognostic value of the miR urine levels. Some studies have already developed a combined expression signature of multiple miRs, which require a powerful validation strategy. More importantly, independent validation studies are needed to validate these results and evaluate the performance and prognostic power of the signature. Pignot *et al*.^23^ used three miRs to develop a molecular signature for BC with promising results. In the future, developing a new molecular signature using diverse miRs and then identifying their efficacy may also be useful.

In conclusion, our comprehensive systematic review and meta-analysis suggested that miRs, particularly miR-21, miR-143, miR-155, miR-214, and miR-222, could potentially serve as risk stratification markers and even therapeutic targets in BC despite the abovementioned limitations. However, more large scale, multicenter prospective studies with standardized methods and long-term follow up are needed to verify our results.

## Methods

### Search Strategy

This meta-analysis was conducted following the guidelines of the Preferred Reporting Items for Systematic Reviews and Meta-Analyses (PRISMA)^70^.

A systematic literature search was performed in the electronic databases PubMed, Web of Science, and Embase on 15 March 2016 using the following search strategy: (microRNA or miRNA or miR) and (bladder cancer or bladder tumor or bladder carcinoma or bladder neoplasm or urothelial cancer or urinary tract cancer) and (prognosis or prognostic or survival or outcome or mortality). We also manually searched the reference lists of the relevant literature.

### Selection Criteria

The studies were included based on the following criteria: (1) the association of miRs with the prognosis significance in BC should be described; (2) the studies defined the miR cut-off and clearly described the miR measurement; and (3) the studies correlated survival outcomes with a single miR expression. The exclusion criteria were as follows: (1) non-English papers; (2) case reports, letters, commentaries, meeting records, or review articles; (3) sample number fewer than 30 patients; (4) focused on animal models or cancer cells; (5) concerned genetic variation of an miR; (6) calculated HRs based on multiple miRs; and (7) the study lacked sufficient data for obtaining HR and 95% CI. All evaluations were independently performed by three individual researchers to ensure the accurate inclusion of studies. The discrepancies were resolved by discussion. We only retrieved the most informative and recently studied one for further analyses of duplicate studies.

This study was based on published literature. Therefore, ethical approval from ethics committees was not needed.

### Data Extraction

Three investigators independently extracted data from eligible studies using a predefined form. The discrepancies in data extraction were resolved by discussion. The following data were extracted: surname of the first author, publication year, investigated miRs, origin of the studied population, study design, tumor stage, sample size, gender, follow-up time, detecting method, detected sample, cutoff value, and effect estimates, namely, HR of miR expression for OS, CSS, DFS, RFS or PFS, as well as their 95% CI (Table 1). We calculated HRs and their 95% CI based on the methods reported by Tierney *et al*.^68^ if the HR and 95% CI were not directly available.

### Quality Assessment

The quality of the included studies was evaluated using the Newcastle–Ottawa scale, as recommended by the Cochrane Non-randomized Studies Methods Working Group^71^. Each study can be assessed by eight methodology items with a score ranging from 0 to 9. The high scores indicated high quality. We considered studies with scores of 6 or more as high quality for the meta-analysis. Only studies with high quality were included in the further analysis to assure the quality of this meta-analysis.

### Statistical Analysis

Pooled HR with 95% CI was used to evaluate the association of the miR expression with the BC prognosis. An observed HR > 1 implied an unfavorable prognosis for the group with an elevated miR expression. Conversely, an observed HR < 1 implied a favorable prognosis for the group with an elevated miR expression. A heterogeneity test of the pooled HR was conducted using Cochran’s Q test and Higgins I-squared statistic^72^. A p value of less than 0.05 was considered significant. A random-effect model was used when between-study heterogeneity was observed (Cochran’s Q test, p < 0.05); otherwise, a fixed-effect model was used. All statistical analyses were performed using Stata 12.0 software (StatCorp, College Station, TX, USA), and p < 0.05 was considered statistically significant.
